# Trauma, Violence, and Memory in African Child Soldier Memoirs

**DOI:** 10.1007/s11013-020-09668-4

**Published:** 2020-03-02

**Authors:** Stacey Hynd

**Affiliations:** Department of History, University of Exeter, c/o Old Library Mail Room, Prince of Wales Drive, Exeter, EX4 4SB UK

**Keywords:** Child soldier, Memoir, Africa, Humanitarianism, Trauma, Conflict

## Abstract

Child soldiers have been heavily involved in contemporary African warfare. Since the 1990s, the ‘child soldier crisis’ has become a major humanitarian and human rights project. The figure of the child soldier has often been taken as evidence of the ‘barbarism’, dehumanization and trauma generated by modern warfare, but such images can obscure the complex reality of children’s experiences of being part of armed groups during conflict. This article uses the published memoirs of former child soldiers from Sierra Leone, Sudan, Uganda, Eritrea and the Democratic Republic of the Congo to explore the instrumental and discursive nexus between child soldiers, memory, violence and humanitarianism. It assesses how (former-) children combatants remember and recount their experiences of war, and how these narratives can be shaped by humanitarian, literary and/or therapeutic framings. The article argues that these memoirs’ significance lies in their affective truths and what they reveal about children’s experience, and narrations, of war. Former child soldiers engage with, but also challenge, dominant contemporary humanitarian discourses surrounding childhood and violence to develop a ‘victim, savage, saviour, campaigner’ framework for their narratives. The article historically contextualizes the emergence of the ‘child soldier memoir’, before analysing the narratives of recruitment, indoctrination, and violence recounted by these former child soldiers, and their attempts to rework their identities in a post-conflict environment. It explores how former child soldiers narrate suffering and deploy discourses of trauma in their memoirs: some seeking to process wartime traumas, others leveraging their own suffering to position themselves as campaigners for those children still caught in conflict.

## Introduction

Children have historically been heavily involved in conflict, as victims, perpetrators, and witnesses, both in Africa and across the world. Global estimates from the 1990s and early 2000s posit that over 300,000 child soldiers were fighting or had recently been demobilized. An estimated 120,000 of those were African, and indeed for most of the 1990–2000s the iconographic image of ‘the child soldier’ was overwhelmingly African—a small, wild-eyed African boy in ragged clothes, brandishing an AK-47 (CSUCS 2008). Since the 1990s, the ‘child soldier crisis’ has become a major humanitarian and human rights project, from the United Nations Machel Report in 1996 to the Kony 2012 phenomenon and the #BringBackOurGirls outcry over the 2014 kidnapping of 276 schoolgirls by Boko Haram in Nigeria. Dominant humanitarian discourses of child soldiering have framed children’s involvement in war as a problem primarily of contemporary, asymmetric, and highly violent warfare, linked to a breakdown of familial and social child protection mechanisms, and driven by forcible recruitment by military commanders. These discourses have tended to reject child agency and accountability, portraying child soldiers as iconic victims of war (see Brett and McCallin 1994; Machel 1996). The humanitarian appeal of the African child soldier is rooted in how “the brutal existence of a child soldier dovetails neatly with depictions of Africa both as a place of hell and misery and as a continent that, like a child, can be saved” (Mengestu 2007). Humanitarian campaigns have highlighted the abduction and forced recruitment of children, depicting child soldiers as brutalized, traumatized victims of adult abuses, whose recruitment violates norms of both war and childhood and whose rescue requires international action (Brett and Specht 2004). Humanitarian and human rights-based interventions to prevent the recruitment and use of children in armed forces are, however, predicated on a contemporary “transnational politics of age” that enshrines Western-originated, now globalized norms of childhood as a space of innocence, education, and freedom from labour and sexual activity (Rosen 2007:296–298). These norms have been subject to sustained academic critique and are far from reality for many African communities, where local understandings of childhood are based more on social and physical status rather than chronological age and foreground children’s capacity to be active social agents and productive members of a household, sometimes highlighting the potentially disruptive liminality of children and their capacity for action and violence rather than any innate state of innocence (James and Prout 1990; Twum-Danso 2005; Nieuwenhuys 2010; Shepler 2014). The image of the innocent and brutalized child soldier as victim in these contemporary humanitarian campaigns therefore “repeats [a] colonial paternalism where the adult Northerner offers help and knowledge to the infantilised South”, positioning non-governmental organizations as better able to provide for the needs of children than their own families and societies, and pathologizing children’s agency in socially navigating conflict environments (Burman 1994:241; Lee-Koo 2011:735; Pupavac 2001). What both supports and disrupts these humanitarian discourses are the voices and memories of African child soldiers themselves.

When it comes to accessing memories of conflict, the published memoirs of former child soldiers grant international audiences detailed individual insights into African conflicts and societies in a way that has been managed by very few other voices. This article suggests that whilst sometimes problematic, these memoirs can be productive tools in researching modern African conflict because of what they reveal about how contemporary humanitarian discourses and ideas of trauma shape the narration of war memories, and for what they reveal about the agency and resilience of former child soldiers and the quotidian realities of conflict. The memoirs analysed herein are the ten most widely available commercially published texts written by former child soldiers who were involved civil wars across sub-Saharan Africa spanning from the 1980s to the early 2000s. The most famous of the memoirists are Ishmael Beah and Emmanuel Jal. Beah was forcibly recruited into the Sierra Leonean armed forces during that country’s civil war, and was demobilized into a UNICEF rehabilitation centre in Freetown before relocating to America and writing his memoir, *A Long Way Gone*, which became an acclaimed bestseller and was even sold in Starbucks coffee shops (2008). Beah has since become a UNICEF advocate for war-affected children, and a novelist. Emmanuel Jal was recruited into the Sudanese People’s Liberation Army [SPLA], surviving life in Pinyudu refugee camp and several military engagements before being taken to Nairobi by Riek Machar’s English wife Emma McCune. A 2008 documentary about his life, *War Child,* was followed by the publication of his memoir (2009). Jal has since gained international renown as a political activist, rap artist, actor, and founder of the charity Gua Africa. This article also addresses the experiences of two (South) Sudanese ‘Lost Boys’, Deng Adut and Cola Bilkuei, who moved from being SPLA child soldiers to refugees, before settling in Australia (Adut and Mckelvey 2016; Bilkuei 2013). Insights into child soldiering in the Democratic Republic of the Congo [DRC], like South Sudan an ongoing zone of child recruitment, are provided by the memoirs of Lucien Badjoko and Junior Nzita Nsuami, two *kadogos* (little soldier; child soldier) who served in the Congo Wars (Badjoko and Claren 2005; Nzita Nsuami 2016). Girls constitute an estimated thirty per cent of child soldiers and their voices are well-represented in memoirs (McKay and Mazurana 2004). China Keitetsi gives the earliest account detailed here, covering her time as one of Yoweri Museveni’s *kadogos* in the Ugandan bush war and after Museveni’s seizure of power in 1986 (2004). From the Lord’s Resistance Army [LRA]’s forced recruitment of children in Northern Uganda, memoirs have been written by Grace Akallo, one of the ‘Aboke Girls’ kidnapped by the LRA in 1996, and by Evelyn Amony, who was abducted as a young girl and forced to become one of Joseph Kony’s ‘bush wives’ (McDonnell and Akallo 2007; Amony 2015). Finally, Senait Mehari provides a contested account of her experiences in a disintegrating Eritrean Liberation Front [ELF] unit in the Eritrean independence struggle against Ethiopia (2006).

This article will put forward two main strands of argument. Firstly, that the historical significance of child soldier memoirs lies not so much in their relating of empirical facts, which are sometimes disputed, but rather in what can be called their affective truths and what they reveal about children’s experiences, and narrations, of war. Secondly, it argues that former child soldiers engage with, but also subtly challenge, dominant contemporary humanitarian discourses surrounding childhood and warfare to develop a ‘victim, savage, saviour, campaigner’ framework for their narratives. The article will open by discussing methodological issues about reading child soldier memoirs historically, before qualitatively analysing the functioning of the affective truths of these texts through their narration of memories of recruitment, indoctrination, combat experience, and their subject’s attempts to rework their identities in a post-conflict environment. It will address notions of ‘victimhood’ and ‘victimcy’ (Utas 2011) by looking at memoirists’ accounts of their ruptured innocence and notions of guilt, and address ideas of the ‘savage’ by exploring how violence is narrated. The article will analyse how these child soldier memoirs sit in tension with dominant humanitarian discourses surrounding the—usually white, Western—humanitarian as ‘saviour’ to war-affected children, and how these former child soldiers utilize their stories to position themselves as campaigners for those children still caught in conflict.

This article will use the term child soldier in line with current United Nations categorization to include any person under eighteen years of age who becomes part of an armed group in any capacity, including as a ‘bush wife’ or conjugal slave (UNICEF 1997): it should be noted however that the vast majority of child soldiers are teenagers, who sees themselves as youths or young adults (Twum-Danso 2005; Shepler 2014). Although humanitarian practitioners today prefer the term ‘child [formerly] associated with armed forces or armed groups’ as less potentially stigmatizing, this article retains the term ‘child soldier’ as it is the terminology most often adopted by the memoirists themselves. For clarity, the term memoir will be used throughout, although some of these texts could also be categorized as auto/biography or life writing. There has been considerable debate over whether notions of post-traumatic stress disorder and biomedical or biopsychosocial framings of ‘trauma’ as formulated in the DSM are properly universal and can therefore be applied outside the Western contexts in which they were developed (Summerfield 2000; Honwana 2006:150–156). Memoirs however are not medical texts. This article is not so much concerned with whether or not, or to what extent, these former child soldiers are traumatized, but rather with how they recount suffering and deploy discourses of trauma in their narratives. As Kleinman argues, medicalizing political violence removes the human context of trauma as the chief focus for understanding violence, and those who suffer it should be understood as social sufferers rather than just patients or victims (Kleinman 1988; 1995). Former child soldiers’ accounts are products both of their individual experiences and memories, and the cultural and societal contexts in which they have grown up, being shaped by collective memories of conflict and displacement in both local African cultures and the West (Kevers et al. 2016). This article posits that the suffering expressed by these memoirists is normative for children who have experienced conflict and fighting, rather than pathological, but that many do narrate behaviours and memories that could be classified as ‘traumatic’. An uncritical application of trauma diagnoses in non-Western societies affected by conflict can relabel social suffering as a pathological condition, creating a form of “psychological imperialism” and stressing victimhood rather than recognizing individual and community resilience (Pupavac 2001; Fassin and Rechtman 2009; Kevers et al. 2016; Good and Hilton 2018:8). Discourses of trauma can also serve to depoliticize and individualize suffering, and medico-humanitarian interventions to alleviate that suffering, leading to a failure to address the structural roots of violence in a society or the global inequalities and political economies that fuel conflict (Lee-Koo 2011; Summerfield 2002). However, the evidence presented by these memoirs suggests that discourses of trauma do hold value for some former child soldiers, either because they feel traumatised by their experiences, and/or they have learned to express themselves within such discourses to better communicate their suffering as a result of their engagement with humanitarian organizations, therapeutic interventions, or commercial publishing (Verma 2012). There is a need for the scholarly analysis of war memories to break away from a simplistic binary relationship between ‘trauma’ and ‘resilience’ which flattens the complexity of human emotions and experiences. For these memoirists, their post-conflict resilience is enhanced through harnessing their own suffering in an attempt to alleviate or prevent the suffering of other war-affected children. Writing their ‘trauma’ and bearing witness to the horrors of war becomes a coping mechanism for these former child soldiers, a moral act of memory, helping to give post hoc reason to their suffering and to assuage the guilt that some of them seem to carry at having survived where their friends and comrades have not.

## Reading Child Soldier Memoirs into/as History

The sub-genre of the child soldier memoir emerged from a cultural nexus of the 1990–2000s boom in autobiography and childhood autobiography, the growth of child rights, human rights and liberal humanitarianism, alongside the growing delegitimization of warfare and the development of humanitarian psychiatry, creating what Fassin and Rechtman term a new “moral economy of trauma” (Douglas 2010; Burman 1994; Barnett 2011; Fassin and Rechtman 2009). Whilst children’s experiences and voices have historically been marginalized within collective and public histories of war and politics, these African child soldier memoirs and auto/biographical voices have been, if anything, over-privileged in the creation of globalized, collective memories of contemporary conflict in Africa.

It is striking that child soldier memoirs regarding conflicts that occurred prior to the 1980s, even those written more recently, do not notably employ discourses of trauma, rights or victimhood. The memories are instead narrated with a focus on the author’s resilience and agency in becoming a disciplined soldier despite their suffering, and on making a ‘good war’ for themselves as far as their situation allowed, particularly in Second World War memoirs (Kolk and Mandambwe 2007). Decolonization-era memoirs tend to pay particular attention to the political mobilization and agency of youth in liberation conflicts (Ferdi 1981). Some memoirists even write of war as being in part a positive experience, when they moved from being marginalized children to feeling empowered, respected, and part of a community, as in Talent Chioma Mundy-Castle’s account of her time as a Biafran child spy during the 1967–1970 Nigerian Civil War (Mundy-Castle 2012). Contemporary child soldier memoirs, however, display a qualitative shift in their politics of memory which are instead enframed by the dominant victimhood-orientated humanitarian, human rights, and trauma discourses which define contemporary accounts of war and violence (Schaffer and Smith 2004).

One of the core paradoxes of commercial auto/biography is that memoirist must be both unique and representative in their experiences (Miller 2001:8). These child soldier memoirs are written by exceptional children-turned-adults: exceptional in that they are literate, were personally supported by Western/international humanitarians, and in that they survived and escaped their conflicts. Many left Africa to seek new lives abroad, moving from soldier to refugee or migrant status, bringing a globalized, expatriate perspective to their accounts, and highlighting the tensions between universal and local norms of childhood, particularly in their experiences of coming of age in a wartime environment (Douglas and Poletti 2016:100). As Deng Adut writes “[i]n the eyes of my culture, I am still a boy. When I should have been going through the rituals of manhood, I was caught in a vicious war. By the time I was returned to my people, I was very much a Westerner. My feet straddle continents, and also the threshold of manhood” (Adut and Mckelvey 2016, loc 77). All the memoirists are writing about childhood wartime experiences through their new perspectives as young adults. Life-cycles are important in the framing of traumatic memories, and these memoirs are very much youth narratives, written by young adults who are seeking to establish their identities, their social status, and to claim a position of power from which their voices can be heard and acknowledged (Clifford 2017). The normative character and moral economy of the world in which a person grows up and learns to perform their identity shapes the life narratives that they tell—but for those whose lives are disrupted as children, particularly as teenagers whose identities are fluid and liminal, their normative character is affected by both war and peace, by their natal cultures, their militarized identities, and their new post-conflict, often expatriated, lives as activists, writers, and professionals.

The writing in memoirs differs markedly from human rights reports that generally balance empirical data with sentimentalized narratives and affective appeals, combining statistics with excerpted, decontextualized, individual testimony, and striking images (Schaffer and Smith 2004). All commercially available memoirs are edited and adapted through the publishing process, but many child soldier memoirs are also co-written, mediated testimonies (Douglas and Poletti 2016:97–98). Badjoko, Jal, Adut, and Mehari’s memoirs were all collaboratively written with Western journalists/authors, the extent of whose input is left unclear. The language varies sharply, from Jal and Beah’s more poetic, fluid prose, honed by their creative talents as rapper and novelist, respectively, to the more fractured, idiomatic language of Keitetsi. Erin Baines, the American academic who edited from a series of interviews Evelyn Amony’s account of her life in the Lord’s Resistance Army as one of Joseph Kony’s wives, notes that when recollecting traumatic stories Evelyn’s narrative became less comprehensible or sounded detached, and Baines therefore deliberately worked towards a written transcription that captured this reaction (2015:xx). Tellingly, the memoirs of Lucien Badjoko, one of Laurent Kabila’s *kadogo* in the Democratic Republic of the Congo [DRC], were co-written with a French journalist, Katia Clarens, after she met him in a DDR [disarmament, demobilization, and reintegration] camp and he informed her “I have written a short script of my life if you are interested” (2005:7).^1^ Badjoko here displays a variant of what Utas terms victimcy tactics: children expressing agency through identifying themselves as powerless victims and appropriating humanitarian tropes to secure funds, resources or protection (Utas 2011; Verma 2012). As Kleinman argues, trauma stories have become a currency that social sufferers of political violence and conflict exchange to claim new status as refugees (Kleinman 1995:177–188). Both Cola Bilkuei and Deng Adut, former SPLA child soldiers from South Sudan who relocated to Australia, learned to craft and commoditize their life stories to support themselves as they transitioned from military life to refugee and immigrant status. Bilkuei began to shape his narrative to his audience when moving between East African countries and refugee camps seeking aid, and first wrote his life story to be ranked in order of need by the UNHCR in Kenya in 1996: “I started to think about telling my story to anyone who would listen, hoping they would give me money in exchange. Not for the first or last time, my life story was becoming my meal ticket. It might be odd, for an Australian, to see your life story as your sole economic asset. But for me and other Sudanese who have little else to sell, it was a natural thing to do” (2013:153). Ishmael Beah was sent to the United Nations in New York as a child soldier representative and was there drawn to the woman who would become his adoptive mother, Laura Simms, because “[s]he said she would teach us how to tell our stories in a more compelling way” to engage with international audiences (2008:196).

When many former child soldiers crafted their texts, they therefore had practice at producing the range of narrative elements that Western readers and editors have come to expect, as well as drawing on oral storytelling traditions from their own cultures (Moynagh 2011:48; Beah 2008:217–218). Unlike human rights reports or truth commission testimonies which are focused on establishing factual accounts of conflict, memoirs, like child soldier novels, often display a marked ahistoricity, eliding context, time and space, and blurring the boundaries between history, memory, and narrative truth: historical and political context to the conflicts they fight in is replaced by an overarching framework of human rights abuses and humanitarian narratives (Coundouriotis 2010). The preferred narrative framework is one of innocence disrupted by war, violence, and trauma, then humanitarian salvation and recovery, with a corresponding disavowal of violence. It is perhaps significant that those memoirs which most closely follow this framework, like Beah and Jal’s, are those which have become the best-selling and most widely lauded by both public and humanitarian sources. Child soldier memoirs are strongly influenced by humanitarian discourses of child soldiering that code children’s involvement in war as an adult-perpetrated human rights abuse caused by social breakdown and hyper-violent, non-rule bound contemporary conflict, and which reject child agency and culpability (see Machel 1996). The memoirs, however, both challenge and support these discourses (Schultheis 2008; Mackey 2013; Mastey 2018). Where they primarily challenge those discourses is in the much higher level of agency they accord themselves—however tactical or circumscribed that agency is—and in the significance of that agency to their survival and salvation, rather than expressing a reliance on external, humanitarian rescue (Honwana 2006:50–51; Drumbl 2012:98–101).

Makau Mutua writes that contemporary human rights narratives about Africa routinely follow a framework of ‘Savage, Victim, Saviour’ (Mutua 2001), detailing violence and establishing victimhood to justify external intervention and rescue. Paraphrasing Mutua, this article argues that child soldier memoirs follow instead a framework of ‘Victim, Savage, Saviour, Campaigner’, establishing child soldiers as victims of war and military recruitment, who enact and experience violence before being rescued—or rescuing themselves—and seeking to help prevent others suffering their fate. All of the memoirists now campaign against the use of child soldiers, and many explicitly state that their narratives were written to assist these campaigns and bear witness to the violence committed against child soldiers: as Emmanuel Jal sings in his song ‘War Child’, ‘I believe I’ve survived for a reason, To tell my story, to touch lives’ (Jal and Davies 2009:257). One of the kidnapped Aboke girls from Northern Uganda, Grace Akallo, rhetorically asks herself why she has survived in the LRA, escaping death in raids, from beatings, and even multiple suicide attempts. She credits God for her survival and eventual escape from captivity, but believes that for survival to have a purpose: “Surely there was a reason I had escaped death. Maybe I might help change the ten-year-old war… God if You let me go back…I will fight for the children who become victims in this war. They cannot speak” (McDonnell and Akallo 2007:113, 125–126). Akallo developed this theme in her testimonies to the UN Security Council and the US House of Representatives, telling the House that what she had faced during her abduction was “beyond fear” and that she was not there to “evoke emotions without action” but to plead for justice and accountability against those who recruit child soldiers (US House of Representatives 2008; UN SRSGCAC 2009). Beah asserts that “my own trauma is a small price to pay to expose what continues to happen to children all over the world” (Beah 2008:3). These memoirs are performative of a new form of identity for many former child soldiers: that of the empowered ‘victim’ turned ‘campaigner’. Where possible, these memoirs should be read paratextually within the context of wider humanitarian coverage of child soldiering and the author’s own websites, interviews, TED talks, and other cultural productions. Nzita Nsuami’s memoir *If My Life as a Child Soldier Could be Told* was presented to the UN mission in the DRC and is prefaced by Leila Zerrougui, Special Representative for the UN Secretary-General for Children and Armed Conflict, who supports Nzita Nsuami in using “his experience for the benefit of many other child soldiers”, with proceeds of the memoir going to his organization Paix pour l’enfance (Nzita Nsuami 2016, first preface). The memoirists have all been active in campaigning against the recruitment of child soldiers and other related rights abuses. They have worked with groups such as UNICEF, Human Rights Watch and the Coalition to Stop the Use of Child Soldiers to voice their campaign appeals and spoken about child soldiering at the United Nations, as well as setting up their own organizations, such as Jal’s Gua Africa, or worked locally with rehabilitation and reintegration projects for former child soldiers and refugees.

## Empirical, Narrative, and Affective Truth in Memoirs

As a body of evidence, child soldier memoirs sit amongst therapeutic writing, testimony, human rights reporting, humanitarian campaigning, coming of age narratives, post-colonial life history, and oral storytelling. As Kate Douglas argues, they are products of, and confrontations with, cultural memory (Douglas 2010:20). Historically speaking, their narratives sit between empirical or forensic truths, narrative or personal truths, and what can be called affective truths. The term affective truth is used here to argue that these narratives are specifically crafted to prompt an emotional response or “empathic unsettlement” in their readers through authoritative accounts of the—purported—reality of child soldier experience, and to thereby generate humanitarian sentiments and actions from readers (LaCapra 2014). This is not to say that this affective truth does not also stem from a genuine emotion that the author needs to process and convey, but rather that in the published memoir this emotion is deployed to convey the veracity of experience and is leveraged to prompt a particular response in the reader.

There are perennial ethical and methodological debates over the ‘truthfulness’ or factual nature of memoirs, particularly regarding accounts like those of Ishmael Beah and Senait Mehari who have come under attack by the media and other former child soldiers in their units for misrepresenting duration of their involvement for or appropriating others’ stories (Sanders 2011:206–207). Beah notes of his time in the Sierra Leonean army that “My mind had not only snapped during the first killing, it had also stopped making remorseful records, or so it seemed” but then also recounts his military experiences in great detail with extensive dialogue, asserting he has a photographic memory (Beah 2008:122, 51). The historical utility of memoirs is not primarily linked to forensic or empirical truths, establishing the facts of what happened, as is the case with human rights reports. But these texts are not simple narrative truths either, nor just an individual’s memories of war. What is most significant is how, and why, former child soldiers feel compelled to relate their memories. Their accounts speak to what Baines terms the “ethical significance” of the events they experienced (Amony 2015:xxi). Childhood autobiographies today are commonly marketed according to their political and sociological worth, their virtue signalled in their promise of didacticism and their exposure of social injustices (Douglas 2010:61). In African child soldier memoirs, this didacticism and exposure operates through the crafting of an affective truth that is framed by notions of violence, suffering, and trauma, contrasting with the innocence and protection that is assumed to be due to children under universal norms of childhood in contemporary geopolitical frameworks (Burman 1994; Mastey 2018). Child soldier narratives and images have become so prominent within contemporary humanitarian iconography precisely because child soldiers violate established generational and moral norms, and complicate notions of child victimhood and apolitical status with the juxtaposition of their imbued innocence and violent action: they are profane figures that disrupt the affective and semiotic apparatus of humanitarian concern (Malkki 2015:2, 8). Humanitarian advocacy has to balance highlighting the danger occasioned by (as well as for) child soldiers to raise awareness with establishing that they are still redeemable in order to convince audiences of the efficacy of intervention, a balance the memoirists help establish.

## ‘Victims’ to ‘Savages’: Innocence, Violence, and Narrative Rupture

The memoirs nearly all take on a chronological, broadly *Bildungsroman* structure stretching from childhood innocence, to the rupture of their entry into an armed group, through the horrors of war to their escape and salvation, and then their struggles to re-adjust to civilian life and eventual empowerment through their personal success and activism. Some open with a nostalgic account of their childhood and the perceived happiness and security of an idyllic family life, with Grace Akallo recalling “[t]he village I knew as a child was a special place. We children felt loved and taken care of…there was never any news of a child being harmed”, cherishing a sense of community and family (McDonnell and Akallo 2007:48–49). Others challenge normative, Western assumptions of childhood innocence as a state of ‘not-knowing’ and demonstrate how the conflict had disrupted their domestic security and happiness even before their recruitment by an armed group as they were exposed to fighting through attacks on their villages (Beah 2008; Amony 10–13). Some memoirists conversely highlight their domestic marginalization and lay blame for their entry into armed groups not just on the soldiers who took them, but also upon their families for failing to protect them. Mehari details the abuse she suffered at the hands of her father, but claims she understands the rationality of his decision to hand her and two sisters over to the ELF to reduce the strain on the family’s limited food supplies and prevent them all starving (Mehari 2006:53). Keitetsi recalls the domestic abuse that drove her to voluntarily seek enlistment with the NRA whilst Amony locates her abduction by the LRA within her father’s failure to pay her school fees, as she was captured after being sent away from school (Keitetsi 2004:52–112; Amony 2015:147).

Humanitarian discourses surrounding child soldiering have focused on forced recruitment and abduction as the primary vectors of recruitment. The 1996 Machel Report for the United Nations, the foundational text for contemporary interventions on child soldier issues, issues a stark denial of voluntary recruitment, asserting that children lack the capacity for such agency: “While young people may appear to choose military service, the choice is not exercised freely”, and is instead shaped by external forces (Machel Report 1996:17). Certainly a number of these memoirs demonstrate the prevalence of forced recruitment and abduction (McDonnell and Akallo 2007:93–4; Amony 2015:16–18; Nzita Nsuami 2016). Bilkuei and Adut were both given up by their families in the face of SPLA coercion as part of village quotas for recruitment, whilst Beah was forced to choose between joining the army at gunpoint or being left without protection or resources (Bilkuei 2013:27–29; Adut and Mckelvey 2016; Beah 2008:106). However, as recent studies have shown, many children do demonstrate limited, tactical agency comparable to that of adult recruits in their recruitment (Honwana 2006; ILO 2003). This is borne out in the accounts of Keitetsi, and of Badjoko who, aged twelve, voluntarily joined the Alliance of Democratic Forces for the Liberation of Congo-Zaire [AFDL] alongside his school friends and recalls how “A fire started burning in my stomach. I think about the films I watch every day on video at home…Schwarzenegger, Norris…I really admire them. I’d like to take up a weapon too…We’ve got to liberate the country from tyranny” (Keitetsi 2004:114; Badjoko and Clarens 2005:18).

Most of the memoirs contain a “narrative trajectory of rupture” (and later restoration) in their moral framework, with the moment of entry into an armed group framed as a destructive form of rebirth.^2^ New recruits were stripped of their old identities and resocialized into the cultures and power hierarchies of armed groups, which were often framed as new ‘family’ units to capitalize on juvenile loyalties and disciplinary structures (Nzita Nsuami 2016:III; Dallaire 2011:102–104; Wessells 2006:57–78). Jal remembers SPLA leader John Garang telling the young recruits at Pinyudu to “[a]lways remember that the gun is your father and your mother now” (2009:96). Processes of military training, indoctrination, and dehumanization generated the moral rupture necessary to inure children to violence and killing, whilst physical violence hardened their bodies to suffering in preparation for war. Discourses of revenge were potent in generating hatred towards the enemy: “visualize the enemy, the rebels who killed your parents, your family, and those who are responsible for everything that has happened to you” (Beah 2008:112). This was sometimes supplemented by calls for regime change, the creation of a better, more equal society—or just personal empowerment and enrichment. Taken together, such processes were often successful in remodelling children into soldiers primed to fight for their new commanders. Badjoko remarks that “I have become someone dangerous and I love it. Yes, I am a killer’ and ‘Power is good. I get a kick from it every day. I like to see people move aside as I go by” (2005:80). For many teenage boys in particular, violence formed an attractive and accessible pathway to manhood, with militarized hegemonic youth masculinities based on the power and social status garnered through displays of force and the skills, discipline, and knowledge developed to become an effective soldier. As such, despite their post-conflict rejection of violence, former boy soldiers frequently reflect back on their training and early combat experiences with an ambivalent pride. As Jal notes before his first attack “I was a man, not a coward. We had AK-47 s. We are brave and strong. We were trained fighters who would win this war” (2009, 101). Activities normatively associated with childhood innocence, like play, surface episodically throughout the memoirs, but usually to highlight the loss or corruption of that innocence (see Stargardt 2006; Mastey 2017). Beah contrasts his happy memories of playing soccer as a boy with the horror of shooting other child soldiers across a soccer pitch, recalling sitting on their corpses to eat their food whilst “blood leaked from the bullet holes in their bodies” (2008:19). Mehari recalls how ‘play’ became denoted as a juvenile activity that older children avoided to signal their transition to militarized identities and superior ‘adult’ status (2006:70). Yet not all children were successfully militarized; for some, their identities remained liminal or compartmentalized, struggling alternatively to lose or to retain their civilian identities and moralities. China Keitetsi, a former *kadogo* with Yoweri Museveni’s National Resistance Army [NRA] in Uganda, noted that “I had come so far but I never seemed to harden. It was strange to see most other children having a kind of lust for killing and torturing…It annoyed me that I always had to feel sorry for others, even the enemy” (Keitetsi 2004:134).

## Violence as a Currency of Legitimacy?

Violence was at the core of child soldier experiences, and for boys was central to the development of militarized masculinities. Badjoko extensively details the importance of physical violence, and its emotional impacts, in recasting his identity from civilian to soldier: “I take shape every day. Brutally. The machine guns rattle—I evolve. A friend whose legs are torn off dies in my arms—I grow up. I torture a prisoner—I advance” (2005b, Acte 2). Former child soldiers typically narrate their first kill as the defining moment in their transition from civilian to military life. Beah similarly gives a graphic account of being ordered to slit a prisoner’s throat as part of a training contest. “The corporal gave the signal with a pistol shot and I grabbed the man’s head and slit his throat in one fluid motion. His Adam’s apple made way for the sharp knife and I turned the bayonet on its zigzag edge as I brought it out. His eyes rolled up and they looked me straight in the eye before they suddenly stopped in a frightful glance…The boys and the other soldiers who were the audience clapped as if I had just fulfilled one of life’s greatest achievements” (2008:125). The narration of such extreme violence serves in these memoirs to highlight the shock of the fall, the rupturing of childhood innocence, indicating the depths of savagery from which these child soldiers need to be recovered. The narrative depiction of killing in modern war memoirs has shifted in line with wider attitudes towards violence and the legitimacy of war, moving from the reticent to the brutally explicit (Bourke 1999). Soldiers have transformed in popular discourses from ‘sacrificial victims’ to ‘crazed killers’ or, increasingly, to ‘traumatised veterans’ (Cobley 1994:91). In African child soldier narratives, they are expected to have been all of the above: child/soldier and savage/victim, highlighting the essential liminality of the child combatant. A new “spectacle of suffering” is driving humanitarian constructions of violence (Foucault 1975). Victimhood has become commodified and mediatized in global discourses (Kleinman 1995:188). Violence in these child soldier memoirs therefore becomes a currency of legitimacy, a currency that establishes the extremes of suffering caused and endured, extremes which are necessary to build a successful narrative of redemption and humanitarian support and thereby gain traction in a crowded market of victimhood, which is a prerequisite for generating subsequent concern and action (Hynd 2018). Beah’s narratives of excessive violence aim to establish the authenticity of his status as both victim and perpetrator, drawing the reader in as a witness, a necessary step in a cycle of disclosure that constructs an identity in order to better provoke an empathetic response: as readers we will not wish to be a ‘saviour’ if we are not sufficiently horrified by the ‘savage’.

This currency of legitimacy is however a gendered framework: for girl soldiers, their legitimacy and authority as memoirists and spokespersons on child soldier issues is claimed not through killing but through their suffering of violence, particularly sexual violence (Hynd forthcoming). However, cultural taboos and silencing around sexual violence shape these narratives: girl soldier memoirs tend to recount their experiences of rape and sexual abuse in a very sparing and matter of fact manner, the detail and the emotion is starkly restrained, and the horror remains largely unspoken (Mehari 2006:125; McDonnell and Akallo 2007:110–112). The narration of trauma is clearly gendered in these accounts. The differently gendered accounts, by male and female memoirists, have a different force of affective truth—the male narratives from what they provide in surfeit, the female from what they suppress and leave implicit.^3^

For many child soldiers, their memories are shaped not just by exceptional moments of violence, but by the quotidian realities of conflict: the daily struggles to survive in harsh environments: Mehari notes that she “could not have cared less who the enemy was. My personal enemies were hunger, thirst, the heat, the rats, the hyenas, the relentless military training and the heavy Kalashnikov that I now had to lug around with me all the time” (2006:85). This chronic suffering weighed severely on many children. As Cola Bilkuei recalled “I felt like an old man, worn down by the life that had been handed to me” (2013:93). The apparent frequency of self-inflicted violence and suicide mentioned in the memoirs is indicative of the emotional distress sustained by many child combatants who were unable to socially navigate the violence that surrounded them (Keitetsi 2004:2; McDonnell and Akallo 2007:125; Jal and Lloyd Davies 2009:63). Whilst some child soldiers regard themselves as powerful actors, others find themselves dehumanized and part of an exploitable infrahumanity, both because they are children and because they feel broken by the violence they had experienced (Mbembe 2003:32–34). Bilkuei recalls his fears of becoming dehumanized by his time in Pinyudo, a refugee camp in Ethiopia where many of the SPLA’s ‘Lost Boys’ were held, “I was afraid… If I stayed there, I knew that the need to survive would turn me into an animal” (2013:76). As Keitetsi writes “many acted like robots that only did what our new creators desired. If we were ‘out of order’ we would be sent to the front line to die, sending our memory into oblivion” (2004:125). Regarding the impact of violence and trauma on memory, most of these memoirs display a particular “acoustic register” which focuses not just on sights, but particularly on sounds and smells (Hunt 2008). Jal and Beah both talk about the “Pictures. Pictures. In my head” that repeatedly flash through their memories, but Jal also notes that as part of these: “My time at the front line taught me just one new thing about war—the worst is when it is over. As the battle falls silent, only the screams of the injured can be heard, and when the guns stop firing and the smell of smoke fades away, the stench of flesh and blood fills the air” (Jal and Lloyd Davies 2009:158, 144). Jal states that he could not eat meat for days after a battle because it reminded him of the smell of burning flesh, whilst another former SPLA *jenajesh* (child soldier) Deng Adut recalls the smell of a battle’s aftermath turning his stomach and never really getting his appetite back (Jal and Lloyd Davies 2009:158; Adut and Mckelvey 2016:loc 1014).

## Trauma, Guilt, and Memory

For many child soldiers, avoidance of painful memories and their triggers becomes a pragmatic, culturally appropriate, and often effective coping strategy for the psychological and emotional trauma sustained in conflict, particularly in societies like Sierra Leone where “social forgetting”, a collective avoidance of relating memories of social suffering, is the preferred memory practice, rather than truth-telling (Shaw 2007; Boothby 2006). Memoirists however forgo that coping strategy when they start to write down their memories and narratives. Contemporary African child soldier memoirs are suffused with discourses of trauma and suffering. It is something of a paradox that theoretical discussions often depict violence and pain as essentially unknowable and unspeakable, but that talking and writing are advocated as therapies to heal the victim/sufferer (Miller 2001:6). There is of course a wider philosophical and methodological question as to how, and whether, trauma can be accurately written. As Scarry has argued, pain and violence ‘unmake[s] the world’, whilst Žižek stresses the difference between factual ‘truth’ and ‘truthfulness’ in narrating violence and trauma where the reported content ‘contaminates’ the manner of reporting (Scarry 1985; Žižek 2009:3–4). Despite concerns over its factual accuracy, Mehari’s account of her life still provides a useful example of the memoir as therapeutic writing, having been written after six years in therapy: “Now that I have written everything down, I am free” (2006:x). Keitetsi was also advised to write her memoir *Child Soldier* as a form of therapy: “I just wrote for the sake of emptying myself of the stones that I could feel breaking my shoulders. This book has helped me to come to terms with my past, and helped me come closer to myself” (2004:ix–x). Most of the former child soldiers cited here seem to have written memoirs at least in part as a way of processing their experiences and coming to terms with themselves. It is worth noting however that the labels ‘trauma’ and ‘traumatized’ are much more frequently applied paratextually in interviews, campaigns, or reviews than in the memoir texts themselves. Ethnographic studies suggest that some former child soldiers learn how to ‘perform’ recovery, adopting learned social scripts and discourses of rehabilitation in order to gain support and community acceptance, which raises questions over the efficacy of therapeutic interventions in rehabilitation programming (Verma 2012). Concerns regarding the universality of trauma diagnoses and talking therapies have led to the increasing use of more culturally relativistic, psychosocial forms of therapy in rehabilitation programmes for demobilized child soldiers, with a focus on art therapy, dance, sport, and group counselling (Honwana 2006:150–156; Boothby 2006; Shepler 2014:67–71).

For many child soldiers, including the memoirists, it was after escape or demobilization, with the resultant dislocation from their militarized lives and survival strategies, that the full impact of their experiences emerged and memories of violence became overwhelming. After Keitetsi escaped from Uganda to South Africa, the UNHCR sent her to a trauma therapist. She recalls from her sessions that “I was so afraid to remember…I was so overwhelmed by emotions…I lost control of myself and everything erupted in my mind at once, twenty-four hours a day” (2004:262). Years afterwards, Keitetsi notes “[d]espite this new freedom…I still feel the abuse and humiliation, scars which my body carries still, scars that sometimes make me feel like washing off my skin” (Keitetsi 2004:xi). This embodiment of pain and violence persists for many. Physical and emotional suffering or trauma informs even the methodology of writing. Beah reveals in a published interview that bookends his memoirs that writing required a “reawakening of happy and painful memories, and a deep exploration of them, regardless of the difficulties, physical, emotional and psychological” (2008:endtext 11). Jal writes in his afterword about how “[i]t has been hard for me to tell my story—even physically painful at times as I’ve freed memories buried deep inside. Sometimes my nose bled uncontrollably or dreams would trap me until I woke up to see war still flashing before my eyes” (Jal and Lloyd Davies 2009:41, 61). Intrusive recollections continued to affect many of the memoirists after their escape from warzones, with some recollections seemingly linked to residual feelings of guilt. Beah tells of how “I would dream that a faceless gunman had tied me up and begun to slit my throat with the edge of his bayonet. I would feel the pain that the knife inflicted as the man sawed my neck. I’d wake up sweating and throwing punches in the air”, recalling earlier descriptions of his own initiation into killing (Beah 2008:149).

There is a methodological question as to exactly how useful memoirs are for histories of emotion: do memoirs as ego-documents accurately reveal the feelings and sentiments that the writers experienced during conflict, or are those feelings recast through the process of remembering and relating, with memoirs becoming simply presentist documents? Certainly within these memoirs, former child soldiers’ memories will doubtless have been reframed by their post-conflict lives and by humanitarian discourses. However, emotions linked to war experience are often sufficiently profound—fear, guilt, rage, despair, hope—to have been firmly imprinted on an individual’s memories. Feelings of guilt are a useful example to highlight here. Moral and legal debates abound over whether or not, or to what extent, child soldiers are culpable for their actions in war: are they victims, perpetrators or both? (Drumbl 2012:102–135). International law and humanitarian actors take the position that child soldiers should not be held accountable for their actions, that they are “deviant products of adult abuse” and lack the requisite legally relevant agency to bear responsibility for crimes they commit (Rosen 2007:297). However, child soldiers’ narratives frequently repudiate the “legal fiction” of the non-violent child, and former child soldiers themselves contend with feelings of guilt, highlighting the complexity of the relationship between culpability and guilt (Sanders 2011:199). Some seem to bear a form of survivor’s guilt, their trauma rooted in the “enigma of survival” as their friends suffered and died, before finding a rationale for their survival in their new identities as humanitarian campaigners and activists (Caruth 1996:58: McDonnell and Akallo 2007:181, 190, 194–195). Others, like Nzita Nsuami, assert a religious basis for their survival and adopt a more explicitly Christian discourse of seeking forgiveness: “I am asking for forgiveness from all those to whom I caused harm with the weapons I was made to carry as a child soldier and may they find my repentance expressed in this piece of writing” (Nzita Nsuami 2016:loc. 37).

The guilt that former child combatants feel for the actions they committed in war is rarely explicitly discussed in the memoirs, but it forms an undercurrent to their narration of the violence they participated in, and the intrusive memories which continue to affect them after demobilization. Struggles with feelings of guilt are more prominent in boy soldiers’ memoirs, due to gendered currencies of legitimacy that for males focus on the perpetration of violence and the need to reject such violence in order to claim ‘civilian’ status. Jal notes that “I feel no guilt because I was a child who took part in in killings as the hatred and sorrow built up over years was released in mob violence. I did not kill in cold blood, I killed in war. But that day has tormented me” (2009:265). For many, violence could be normalized and justified at the time of its enactment as a survival strategy within dangerous warscapes. Deng Adut’s most horrified memories center around the torture, killing, and burning of Didinga tribespeople in retaliation for killing an SPLA soldier. “‘We had killed these people, but it didn’t matter. There was so much death around, it did not matter… ‘We were all dead anyway,’ I thought. It was just that some of us didn’t know it yet” (2016:loc 1014). But when reflecting back upon what Adut terms the “mad morality of childhood” as young adults, wartime actions can be reinterpreted through newly adopted (or recovered) moral registers. Adut recalls the killing of the Didinga tribesmen: “When I was a boy, I had been able to put the visions of their melting faces aside because that atrocity had happened in a time and place of endless violence… I hadn’t taken on the weights of those deaths in Sudan, because there was no suggestion that I should feel the shame of their murder”. When he moved to Australia and became a lawyer, however, Adut accepted that he had been “involved in an outrage” and suffers recurring nightmares about their killing: “I think perhaps the torture and murder dreams are a reminder that I owe a debt to the world that I can never repay but must forever try to” (2016:loc 2519–2525). If anything, discussions of feelings of guilt and shame seem to be less about an affective truth that aims to generate action from the reader, and more an internal struggle for self-acceptance.

## Saviours and Campaigners: Military Demobilization & Activist Re-mobilization

Despite being written within and broadly in support of contemporary humanitarian discourses against children’s participation in war, the memoirs are often critical of humanitarian interventions into African conflicts and the forms of child-saving that have taken place (Douglas and Poletti 2016:102–104). Accounts from ‘Lost Boys’ in SPLA and refugee camps in Ethiopia recount in great detail the hardships of life in these camps and the minimal amount of humanitarian aid they actually received. Jal in particular recounts how the SPLA manipulated the *khawajas* (Westerners, aid workers) to secure more supplies, but also later reflects on the structural and racial inequality of global humanitarian actions (Jal and Lloyd Davies 2009:70–73, 181, 249–252; Adut with Mckelvey 2016:loc 1393). Jal, Beah, and Adut were all helped to relocate away from warzones by individual Western women, and as such the memoirs promote the importance of the individual—usually white—saviour figure who breaks humanitarian norms of neutrality to care for the child as a surrogate parent or alternative family member, going beyond regulations to find practical ways to support the former child soldier: a potentially empowering affective truth for the reader who sees the difference one person’s actions can make. But, for the memoirists, these interventions must be based on children’s emotional and material needs, not those of the reader/donor as is common in contemporary humanitarian discourses (see Chouliaraki 2012). Nearly all of the memoirs express a desire for more, and better, intervention that is responsive to children’s varied needs. Tellingly, the memoir that most explicitly draws on humanitarian discourses of trauma, Nzita Nsuami’s account of his life in the DRC, does so to assert that existing rehabilitation programs for child soldiers fail to adequately address children’s needs for therapy and counselling and to call for more such resources, a call supported in the foreword by the UN Special Representative on Children in Armed Conflict (Nzita Nsuami 2016:xx). Conversely, Adut makes the point that he wanted strong men whom he could respect and who could provide him with discipline to help him rehabilitate and assimilate to his new life in Australia rather than “well-intentioned, but painfully underprepared therapists” (Adut with Mckelvey 2016:loc 1618).

The memoirs confirm the importance of gender-sensitive rehabilitation and reintegration, for both male and female soldiers (MacKenzie 2012; Shepler 2014). Jal recalls how after he had been taken out of the SPLA by Riek Machar’s wife Emma McCune to live with her in Nairobi: “It made me angry when she said [that he was too young to be a soldier]. I wasn’t a boy. I was a soldier, and at night my dreams haunted me more than they ever had…Sometimes Emma would try to cuddle me, but I didn’t like it. She was a woman, who should not see my fear” (Jal and Lloyd Davies 2009:88). Militarized identities for boy soldiers are strongly linked to wartime ideas of hegemonic masculinity and attaining ‘adult’ status and power, making demobilization a disempowering rupture for many. In Beah’s account, being repeatedly told “[i]t is not your fault” by civilian workers in the DDR camp antagonised boys who resented the implied lack of agency, and he only slowly came to adopt the humanitarian perspective of himself as a ‘victim’ (Beah 2008:140). Girl soldiers meanwhile, particularly those who have experienced sexual violence and become mothers, face high risks of family and community rejection, and often require additional healthcare, childcare, and vocational training for self-sufficiency (Amony 2015; MacKay and Mazurana 2004).

The memoirs also complicate humanitarian discourses of salvation in the significance they reveal of children’s own agency in exiting armed groups. Seven of the ten memoirists make the decision to escape or demobilize, often at great personal risk. Like many other LRA abductees, Grace Akallo escaped from an LRA camp in Sudan during a battle with Ugandan forces, deciding “it was time to live” and leading other scared and starving children in a trek across dangerous territory back towards Uganda (McDonnell and Akallo 2007:126, 140–141, 156–162, 173–181). The fortitude, resilience, and capacity for tactical thought that was crucial to the children’s survival as soldiers also helped them exit military life and shaped their post-conflict identities, aiding their determination to become activists and campaigners and help save other war-affected children.

## Conclusions

So what then do the published memoirs of former child soldiers from various African warzones reveal about the relationship between memory, trauma, and conflict? These memoirs do reveal significant details about what happened in their respective conflicts, especially about the lived realities of war for child combatants, providing a useful corrective to the often decontextualized narratives excerpted in news or human rights reporting (Moynagh 2011:39). But more significantly, their memoirs are representative of a desire to be “both a model and symbol” of the possibility of rehabilitation and reintegration for other child combatants and war-affected children (Nzita Nsuami 2016:loc. 49, 1028). These memoirs also form part of the authors’ post-conflict coping mechanisms to deal with the mental and emotional impacts of their war experiences. For some, the act of writing processes war-time trauma, aiding in the recovery of lost or submerged memories that can bolster the (re-)establishment of their civilian identities. For others, writing memoirs serves to leverage their suffering towards efforts to ‘save’ other war-affected children, bringing post hoc reason to their ordeals and purpose to their survival. These memoirs are products of contemporary global rights agendas and humanitarian discourses (and commercial publishing agendas) but they successfully highlight the interrelation and coincidence of suffering and resilience, trauma, and agency, and how despite the extremity of some of these memoirists’ distress, they have still been able to survive, and move beyond simple narratives of victimhood to play an active role in society and seek to help others. On a final level then, these memoirs are crafted to impart particular affective truths about conflict with the aim of generating empathy and action. The ‘victim, savage, saviour, campaigner’ narrative framework highlights the rupture and recovery in their moral values, drawing the reader in by revealing the physical and psychological horrors of child soldiers’ lives in war, which particularly focus on their recruitment, indoctrination, and experiences of violence. The narrative framings of the texts also highlight the role of violence as a gendered currency of legitimacy in humanitarian campaigns and memoirs, and its importance in generating emotional reactions in readers with the hope of thereby boosting engaged activism. Considering current concerns about the rise of ‘clicktivism’, as a result of digital social media-led ‘post-humanitarian’ campaigns like Kony 2012 and #BringBackOurGirls, and how they de-emotionalize humanitarian engagement and perpetuate a political culture of narcissism among Western donor-consumers, limiting effective action, perhaps then the affective truths revealed by these memoirs and the voices of their African authors can, and should, take an increased role in speaking out against the targeting and exploitation of children in war, and other related social ills (Chouliaraki 2012).

## Footnotes

1 There are two versions of Badjoko’s memoirs publicly available: the 2005 book and a version available at http://www.grands-reporters.com/J-etais-enfant-soldat.html. All translations mine.

2 Many thanks to Nicholas Stargardt for this point. Notably, in the ongoing trial of Dominic Ongwen, who was abducted as a young boy by the LRA only to become a brigade commander within the group and is now facing war crimes charges at the International Criminal Court, his defence team is building their case around Ongwen’s ‘arrested childhood’, suggesting that his normative moral development was halted at the moment of his abduction and thereby claiming diminished responsibility for his wartime actions. Many thanks to Tim Allen for this information. See International Criminal Court, *The Prosecutor v. Dominic Ongwen*, ICC-02/04-01/15. https://www.icc-cpi.int/uganda/ongwen.

3 Accounts of girls as perpetrators of violence are largely absent from published memoirs and humanitarian discourses, as such accounts challenge both childhood and gender norms.
